# The creation of autotetraploid provides insights into critical features of DNA methylome changes after genome doubling in water spinach (*Ipomoea aquatica* Forsk)

**DOI:** 10.3389/fpls.2023.1155531

**Published:** 2023-04-14

**Authors:** Yuanyuan Hao, Xiao Su, Wen Li, Lin Li, Yu Zhang, Muhammad Ali Mumtaz, Huangying Shu, Shanhan Cheng, Guopeng Zhu, Zhiwei Wang

**Affiliations:** ^1^Key Laboratory for Quality Regulation of Tropical Horticultural Crops of Hainan Province, Sanya Nanfan Research Institute, Hainan University, Sanya, China; ^2^Key Laboratory for Quality Regulation of Tropical Horticultural Crops of Hainan Province, School of Horticulture, Hainan University, Haikou, China; ^3^Hainan Yazhou Bay Seed Laboratory, Sanya, China

**Keywords:** water spinach, autotetraploid, DNA Methylation, phenotypic variation, differentially methylated region

## Abstract

Water spinach (*Ipomoea aquatica* Forsk) is an essential green leafy vegetable in Asia. In this study, we induced autotetraploid water spinach by colchicine. Furthermore, DNA methylation and transcriptome of tetraploid and diploid were compared using Whole Genome Bisulfite Sequencing (WGBS) and RNA-sequencing techniques. Autotetraploid water spinach was created for the first time. Compared with the diploid parent, autotetraploid water spinach had wider leaves, thicker petioles and stems, thicker and shorter adventitious roots, longer stomas, and larger parenchyma cells. The whole genome methylation level of the autotetraploid was slightly higher than that of the diploid. Compared with the diploid, 12281 Differentially Methylated Regions (DMRs)were found in the autotetraploid, including 2356 hypermethylated and 1310 hypomethylated genes, mainly enriched in ‘Arginine and Proline metabolism’, ‘beta − Alanine metabolism’, ‘Plant homone signal translation’, ‘Ribome’, and ‘Plant − pathgen interaction’ pathways. Correlation analysis of transcriptome and DNA methylation data showed that 121 differentially expressed genes undergone differential methylation, related to four pathways ‘Other types of O-glycan biosynthesis’, ‘Terpenoid backbone biosynthesis’, ‘Biosynthesis of secondary metabolites’, and ‘Metabolic paths’. This work obtained important autotetraploid resources of water spinach and revealed the genomic DNA methylation changes after genome doubling, being helpful for further studying the molecular mechanism of variations caused by polyploids of the *Ipomoea* genus.

## 1 Introduction

Polyploids are organisms that contain more than two complete chromosome sets in the same nucleus, which is frequent in nature and plays an important role (Hegarty et al., 2013; Tayalé and Parisod, 2013; Ren et al., 2018). All angiosperms have experienced at least one whole-genome duplication (WGD) event in their evolutionary history, thus, all plants are paleopolyploids (Ahmad and Anjum, 2018; Zhang et al., 2019). The genome doubling event could be either an autopolyploidy (a condition with more than two genomes of the same species) or an allopolyploidy (a condition in which complete genomes of two or more species combine) (Madlung and Wendel, 2013).

Homologous polyploids have double the number of chromosomes and a similar DNA sequence to their diploid parents. Polyploidy has significant effects on plant morphology, such as increased the general vigor and size of various plant parts or structures. Such characteristics are generally referred to as gigantism (Allario et al., 2011; Dai et al., 2015). However, dwarf phenotype caused by polyploidy has also been reported in some plants (Ma et al., 2016; Wang et al., 2018; Wen et al., 2022). It is speculated that the expression level of DEGs was changed after chromosome doubling, which further led to phenotypic changes. This speculation still lacks more evidence support. In addition, polyploidy potentiates plant tolerance to abiotic and biotic stresses (Tossi et al., 2022), such as increased resistance to cold and drought (del Pozo and Ramirez-Parra, 2014; Syngelaki et al., 2021; Abdolinejad and Shekafandeh, 2022).

Polyploidization induces epigenetic changes, including DNA methylation that can be stably inherited in allotetraploid and reversible during genome separation and merger in allohexaploid (Comai, 2005; Chen, 2007). DNA methylation can occur in multiple genomic regions and dinucleotide contexts, including CpG, CHH, and CHG (H = A, T, or C) contexts (Seymour and Becker, 2017). It is involved in many biological processes, including transcription, replication, DNA repair, gene translocation, and cell differentiation (Vanyushin, 2006; Vanyushin and Ashapkin, 2011; Gupta and Salgotra, 2022). Methylation variation in response to ploidy levels has been widely detected (Xu et al., 2009; Salmon and Ainouche, 2010; Xu et al., 2012; Xiang et al., 2023). Previous studies have focused on allopolyploids due to they are widespread and remain an important speciation mechanism (Qiu et al., 2020; Qin et al., 2021). Changes in DNA methylation (including small RNAs) in allotetraploids may affect gene expression and phenotypic variation (Jiang et al., 2021). Although there is increasing methylation as a direct reaction to autologous polyploidy have rarely been reported. Thus little is known about the physiological and molecular mechanisms underlying autopolyploid adaptations (Zhang et al., 2015; Xiao et al., 2022).

Water spinach is an annual or perennial herbaceous plant of the *Ipomoea* genus with heat stress adaptability, both aquatic and terrestrial. In this study, DNA methylation maps of autotetraploid water spinach and diploid water spinach were constructed, and DNA methylation levels, distribution and gene expression were compared. It was found that the genome-wide methylation level of the autotetraploid water spinach was slightly higher than that of the diploid, DNA methylation was involved in regulating gene expression after genome doubling. The study was designed to evaluate the relationship between DNA methylation and phenotypic differences of autotetraploid.

## 2 Materials and methods

### 2.1 Plant material and autotetraploid induction

Water spinach germplasm ‘HNUWS003’ (2n = 2x = 30) was used as the diploid parent. Polyploidy was induced by two methods: The first method was sterile tissue culture (Touchell et al., 2020). The young stem segments of ‘HNUWS003’ with 2-3 internodes were disinfected (70% ethanol, 30 s, 20% NaClO, 20 min) and inoculated in MS medium to produce sterile seedlings. After a large number of sterile seedlings are obtained through cutting propagation, the stem segment with a length of 2.0 ~ 3.0 cm and one internode was placed in 0.25% colchicine solution for 24 hours, then washed with sterile water three times, and finally inoculated on MS medium at the growth temperature of 24 ± 2 °C and the light duration of 16 h/d (1.5 ~ 2.5 Klux). The surviving seedlings with 15 cm were subcultured for 5 times (Nassar et al., 2008).

The second method consisted of a liquid culture. About 15 cm long seedlings of ‘HNUWS003’ with apical bud were cut and soaked in 0.15% colchicine for 18 hours. To ensure the production of adventitious roots on time, stem segments about 2cm out from the cut end cannot be soaked. Some growth points in the leaf axil would sprout new buds, and when they grew into a 15cm seedlings, these seedlings were used for cutting propagation for another 5 times. Liquid culture improved the method of polyploid induction and simplified the induction process. Subsequent analysis was based on autotetraploid water spinach produced by liquid culture.

The seedlings for transcriptome and DNA methylation analysis are propagated by cutting in a growth chamber with a photoperiod of 16 h light (30°C)/8 h dark (28°C). After 20 days of growth, fresh leaves were sampled with three biological replicates, frozen in liquid nitrogen, and stored at -80°C before DNA methylation and RNA-seq analysis.

### 2.2 Paraffin analysis of leaf and root

Leaves and roots of diploid and tetraploid plants were prepared as paraffin sections. The samples were fixed in FAA fixation solution (5 ml 38% formaldehyde +5 ml glacial acetic acid +90 ml 50% ethanol, 1:1:18 by volume) for 24 hours. Then the samples were dehydrated with gradient alcohol, 75% alcohol for 4 hours, 85% alcohol for 2 hours, 90% alcohol for 2 hours, 95% alcohol for 1 hour, anhydrous ethanol I for 30 min, anhydrous ethanol II for 30 min, alcohol benzene for 5~10 min, xylene II for 5~10 min, 65°C melting paraffin I for 1h, 65 °C melting paraffin II for 1h, 65°C melting paraffin III for 1 hour. The wax-soaked tissue was embedded in the embedding machine, and the section thickness was 4μm. Finally, the paraffin sections were stained with safflower O staining solution and plant solid green staining solution, then observed under a microscope and photographed (Hewitson et al., 2010). The characteristics were measured using Image J software.

### 2.3 Root morphology

The root of water spinach was selected and cleaned with sterile water for the subsequent observation of root morphology. An imagery scan screen (Epson Expression 11000XL, Regent Instruments, Canada) was used for root scanning. WinRHIZO 2003a software (Regent Instruments, Canada) was used for root image analysis (Altaf et al., 2020).

### 2.4 Scanning electron microscope analysis

For SEM preparation: fresh leaves of water spinach were cut into squares of about 1cm^2^ and immediately put into the electron microscope fixing solution for 2h. The fixed samples were rinsed with 0.1M PB (pH 7.4) for 3 times, 15 min each. The tissue blocks were transferred to 1% OsO4 and placed at 0.1 M PB (pH 7.4) at room temperature for 1-2 hours. After that, the tissue blocks were washed three times at 0.1M PB (pH 7.4) for 15 min each. The dehydration was performed using a graded series of alcohol-isoamyl acetate concentrations for 15 minutes each. The samples were put into the critical point dryer (Quorum K850) for drying and sputter-coated with gold for 30s by lon sputtering apparatus (Hitachi MC1000). Finally, the images were observed under the scanning electron microscope (Hitachi, SU8100) (Pathan et al., 2008).

### 2.5 Transmission electron microscope analysis

For TEM preparation, fresh leaves were cut into squares of about 1cm^3^, the preparation of slices was finished after fixation, room temperature dehydration, resin penetration, embedding, polymerization, ultra-thin sectioning, and staining. and finally observed and photographed under a transmission microscope (Hitachi, HT7800) (Kuo, 2014).

### 2.6 Flow cytometry analysis

About 0.5cm² of fresh water spinach roots were placed in the petri dish, and then 400μl nuclear lysate were added. The roots were quickly chopped with a sharp blade to facilitate the extraction of complete nuclei. Then, the sample was filtered (30μm nylon filter) and centrifuged. 1600μl DAPI staining solution was added for 10min. The ploidy identification was performed using Sysmex CyFlow^®^ Ploidy Analyser analyzer at a rate of 0.5-2μl/s (Dpooležel et al., 1989).

### 2.7 DNA extraction and qualification

Genomic DNA was extracted using a Plant Genomic DNA Purification Kit. DNA purity was checked using NanoPhotometer^®^ spectrophotometer (IMPLEN, CA, USA). DNA concentration was measured using Qubit^®^ DNA Assay Kit in Qubit^®^ 2.0 Flurometer (Life Technologies, CA, USA).

### 2.8 WGBS Library construction, data filtering, and reads mapping

A total amount of 100 ng genomic DNA spiked with 0.5 ng lambda DNA was fragmented by sonication to 200-300 bp with Covaris (S220). These DNA fragments were treated with bisulfite using EZ DNA Methylation-GoldTM Kit (Zymo Research), and the library was constructed by Novogene Corporation (Beijing, China). Subsequently, pair-end sequencing of sample was performed on Illumina platform (Illumina, CA, USA), and finally generated 150bp paired-end reads. The raw reads sequences were filtered by fastp (fastp 0.20.0), and the remaining reads were counted as clean reads. FastQC was used to perform basic statistics on the quality of clean data reads. Bismark software (version 0.16.3) (Miura et al., 2012) was used to perform alignments of bisulfite-treated reads to a reference genome (-X 700 –dovetail).

### 2.9 Methylation level analysis

Methylated sites were identified with a binomial test using the methylated counts (mC), totals count (mC+umC), and the non-conversion rate (r). Sites with FDR-corrected p-value<0.05 were considered as methylated ones. To calculate the methylation level of the sequence, the sequence was divided into several bins with a bin size of 10 kb. The sum of methylated and unmethylated read counts in each window was calculated. The methylation level (ML) for each window or C site showed the fraction of methylated Cs. The calculation formula was as follow: ML=mC/(mC+umC).

### 2.10 Differentially methylated analysis

Differentially methylated regions (DMRs) were identified using the DSS software, according to the following principles: The proportion of different loci with P value less than 1e-05 was more than 50% of the region, the number of regional loci was more than 3 and the length was more than 50. If the distance between two DMRs was less than 100bp, the two regions were merged. Based on the distribution of DMRs in the genome, DMR-related genes were classified as DMR genes: gene body (from TSS to TES) overlap DMRs, or DMR promoter-genes: promoter regions (2 kb upstream from TSS) overlap DMRs.

### 2.11 GO and KEGG enrichment analysis

The gene ontology (GO: http://www.geneontology.org/) analysis was conducted using GOseq software, and KOBAS (3.0) software was used to detect the statistical enrichment of genes in KEGG (KEGG: http://www.genome.jp/kegg/) pathway.

### 2.12 RNA-seq analysis

The total RNA was extracted from samples, and the mRNA library of each sample was constructed and sequenced in the Illumina platform. Raw data were firstly processed and then clean data (clean reads) were obtained by removing reads containing adapter, reads containing ploy-N and low-quality reads from raw data. Clean reads were aligned to the reference genome using Hisat2 v2.0.5. FPKM of each gene was calculated based on the length of the gene and the reads count mapped to this gene. Differential expression analysis of two conditions/groups (two biological replicates per condition) was performed using the DESeq2R package (1.20.0). Genes with an adjusted P-value ≤0.05 found by DESeq2 were assigned as differentially expressed.

## 3 Results

### 3.1 Phenotypic variation after colchicine treatment

**Figure 1:**
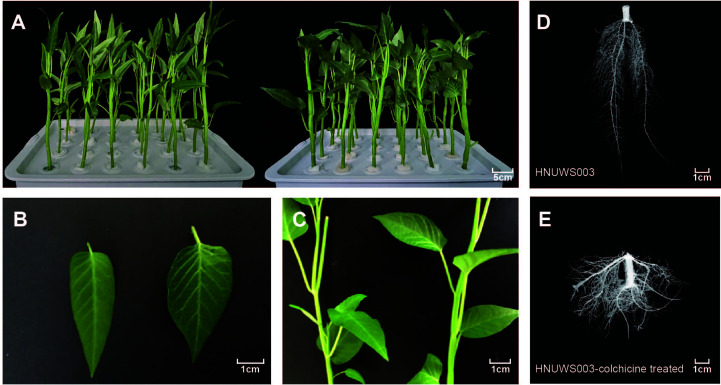
Morphological comparison of colchicine treated water spinach and its diploid parent. **(A–C)** Morphological comparison (**A**: plants; **B**: leaf; **C**: stem; left: HNUWS003; right: HNUWS003-colchicine treated). **(D)** 2X water spinach root morphology. **(E)** colchicine treated water spinach root morphology.

**Figure 2:**
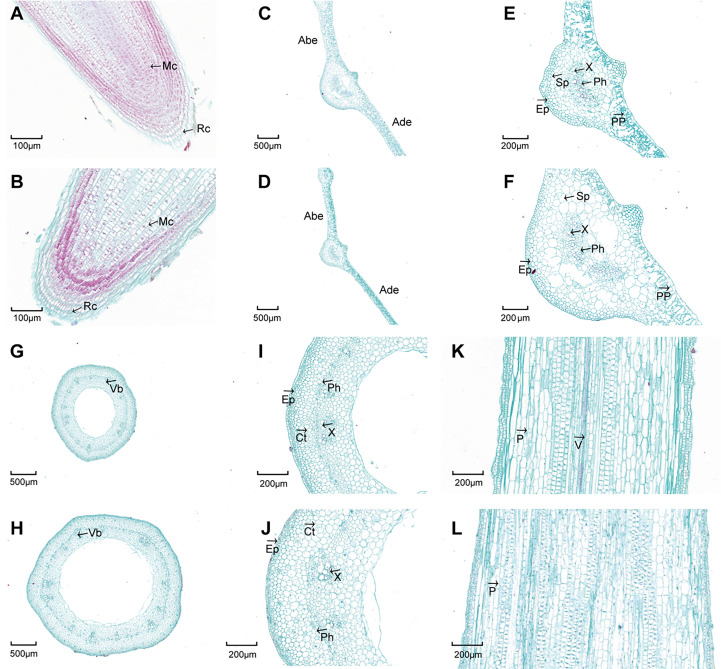
Histological observations between diploid and colchicine treated water spinach. **(A)** Longitudinal-section of 2X water spinach root tips. **(B)** Longitudinal-section of colchicine treated water spinach root tips. **(C)** Cross-section of 2X water spinach leaf (×2.0). **(D)** Cross-section of colchicine treated water spinach leaf (×2.0). **(E)** Cross-section of 2X water spinach leaf (×6.0). **(F)** Cross-section of colchicine treated water spinach leaf (×6.0). **(G)** Cross-section of 2X water spinach stem (×2.5). **(H)** Cross-section of colchicine treated water spinach stem (×2.5). **(I)** Cross-section of 2X water spinach stem (×5.0). **(J)** Cross-section of colchicine treated water spinach stem (×5.0). **(K)** Longitudinal-section of 2X water spinach stem. **(L)** Longitudinal-section of colchicine treated water spinach stem. (Rc, Root cap; Mc, Meristome cells; Ade, Adaxial epidermis; Abe, Abaxial epidermis; PP, Palisade parenchyma; Sp, Spongy parenchyma; Ph, Phloem; X, Xylem; Ep, Epidermis; Vb, Vascular bundle; Ct, Cortex; V, Vessel).

**Figure 3:**
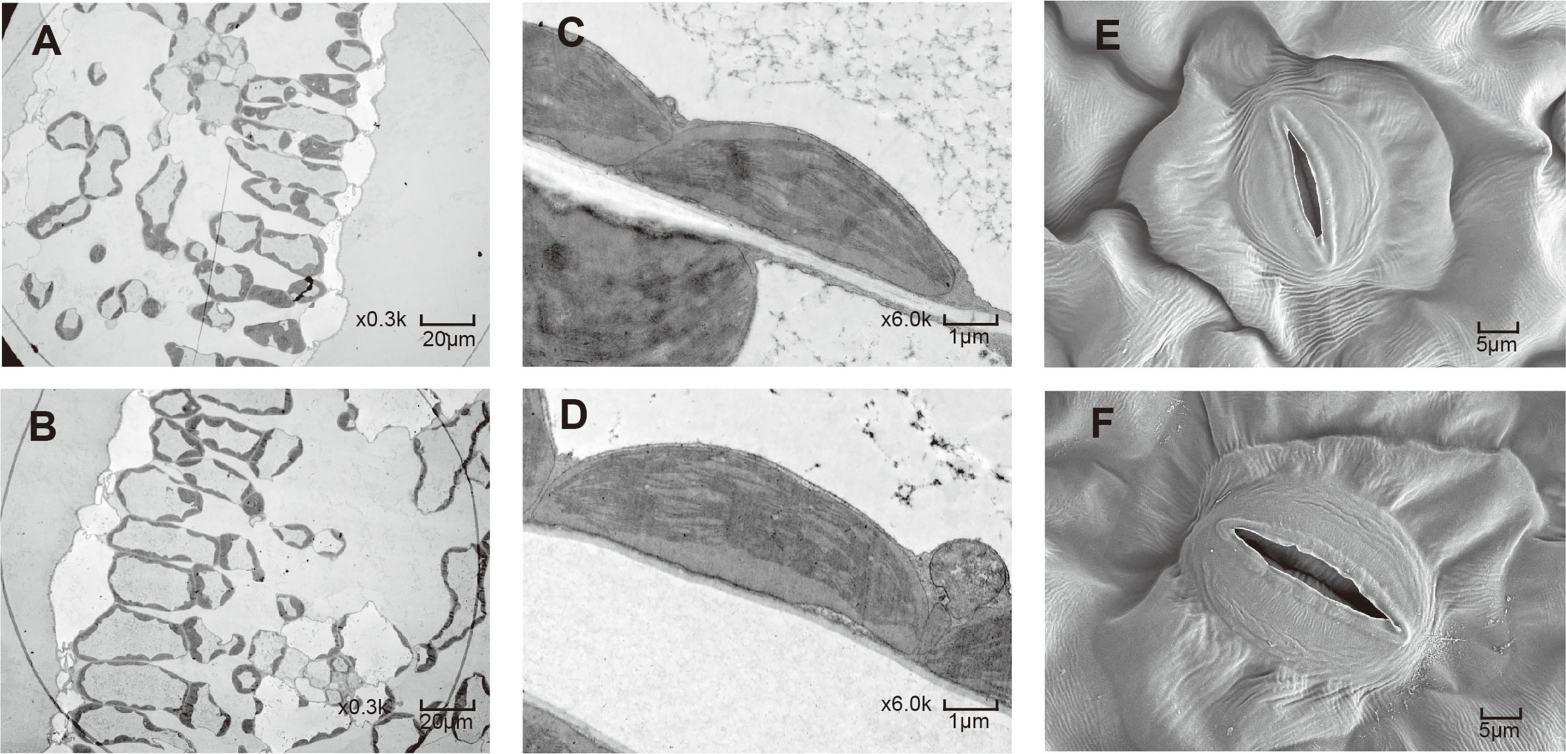
Electron microscope observation. **(A)** 2X water spinach cells under TEM. **(B)** colchicine treated water spinach cells under TEM. **(C)** 2X water spinach chloroplasts under TEM. **(D)** colchicine treated water spinach chloroplasts under TEM. **(E)** 2X water spinach stomata under SEM. **(F)** colchicine treated water spinach stomata under SEM.

**Figure 4:**
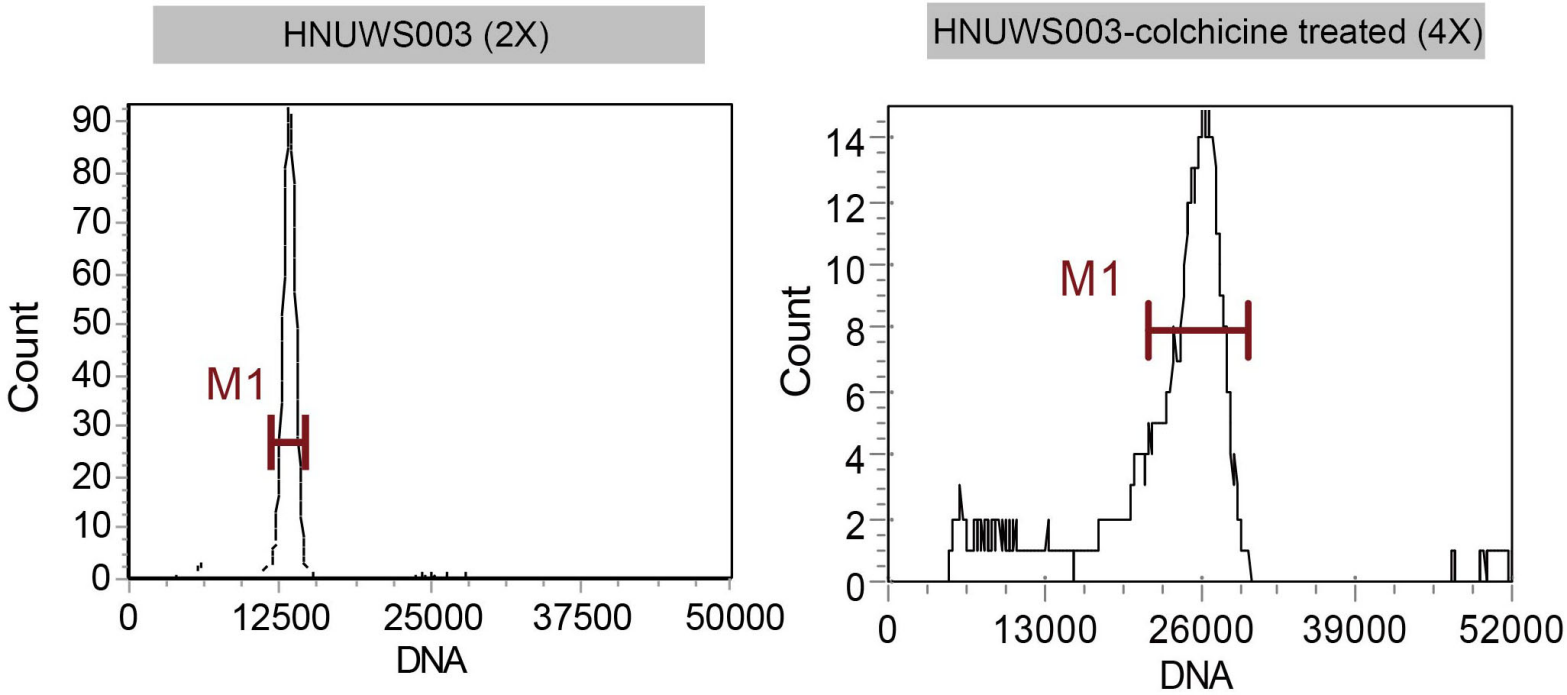
Histograms of flow cytometry finding for water spinach.

After colchicine treatment, some seedlings had no morphological differences from their parents, while some seedlings showed leaf widening characteristics. The seedlings with broader leaves were selected for propagation and named ‘HNUWS003-colchicine treated’ for comparison with the diploid parent (‘HNUWS003’). Here, ‘HNUWS003-colchicine treated’ water spinach has bigger, thicker leaves and stronger stems than the diploid parent. Also, the adventitious roots were thick and shorter (**Figure 1**). Histological observations showed that the leaves of water spinach plants treated with colchicine were thicker, with larger spongy parenchyma cells and palisade parenchyma cells (**Figures 2C–F**; **Table S1**), the diameter and hollow area of stem increased (**Figures 2G–L**; **Table S1**). In addition, the diameter of the roots increased after colchicine treatment and they had larger meristematic cells (**Figures 2A, B**; **Table S1**). TEM revealed colchicine treated water spinach plants has lager cells and larger chloroplasts than diploid parents. Furthermore, SEM showed that the stoma of colchicine treated water spinach was slightly larger than that of diploid (**Figure 3**; **Table S1**). These results indicated that colchicine treated water spinach plants showed obvious phenotypic variation compared with its parents. Flow cytometry was used to investigated the ploidy of colchicine-treated water spinach and control diploid water spinach. The result showed that the nuclear DNA content of the control diploids had a main flow cytometry peak at 12800, while the nuclear DNA content of induced water spinach showed that a main peak at channel 26000, so it can be considered as tetraploid (**Figure 4**).

### 3.2 Genome-wide DNA methylation

To investigate the mechanism of DNA methylation on genome replication, the WGBS sequencing analysis was performed on autotetraploid water spinach and its diploid parent. A total of 105.05 G raw bases were generated, and 93.34 G clean bases were obtained after filtering, with Q20 above 96% and Q30 greater than 89%. Genome mapping analysis revealed 66.22%, 62.61%, and 64.76% clean reads from the three diploid biological replicates, and 67.19%, 67.96%, and 66.61% clean reads from the three autotetraploid biological replicates that were mapped to the reference genome (**Table S2**). The average read depths for diploid and autotetraploid water spinach were 14× and 15.7×, respectively (**Table S3**). C site coverage statistics showed that most cytosine aligned to the CHH context of the genome (**Table S4**).

### 3.3 Methylation site analysis of different ploidies water spinach

**Figure 5:**
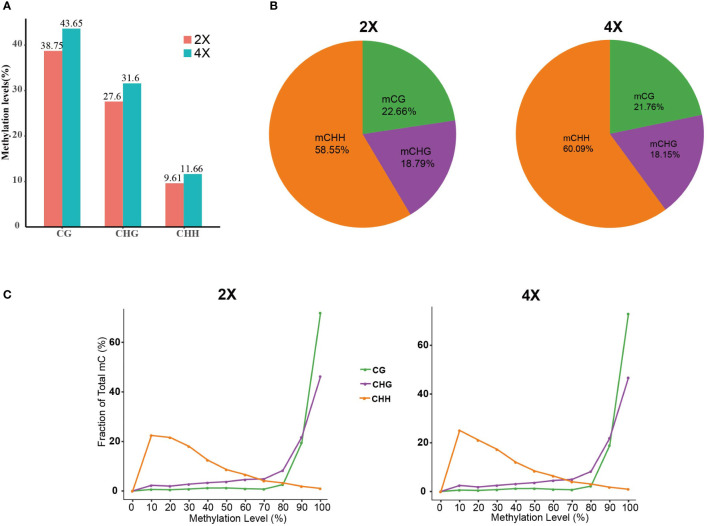
The distribution of methylated C sites. **(A)** Methylation levels in different contexts. **(B)** Map of the proportional distribution of methylated C sites. **(C)** Map of methylation site level distribution.

The percent of methylated C sites in various contexts was determined through methylation site analysis. The result indicated that the mC (methylated C sites) percent in autotetraploid water spinach was higher than that of diploid. In addition, the percent of methylated C sites was the highest in the CG context with about 40%, while the mCHH was the lowest at around 10% (**Table 1**; **Figure 5A**). Among these mCs, more than half of them were found in CHH, then CG, and CHG, which may be due to the majority of C sites in the CHH context (**Figure 5B**).

**Table 1:** Genome-wide methylation C site ratio.

Samples	mC (%)	mCpG (%)	mCHG (%)	mCHH (%)
2X iaq.-1	15.77%	38.52%	27.39%	10.40%
2X iaq.-2	15.05%	38.52%	27.52%	9.43%
2X iaq.-3	14.86%	39.21%	27.88%	9.01%
4X iaq.-1	18.68%	45.25%	32.89%	12.30%
4X iaq.-2	16.70%	40.03%	28.77%	11.16%
4X iaq.-3	18.15%	45.66%	33.12%	11.50%

The methylation level was calculated from the identified methylation sites. mCG and mCHG sites had a significant degree of methylation, with a methylation level of >80%. While mCHH sites had a wide distribution of methylation levels, mostly with slightly methylation (10-30%) (**Figure 5C**).

### 3.4 DNA methylation patterns in different ploidies water spinach genomic regions

**Figure 6:**
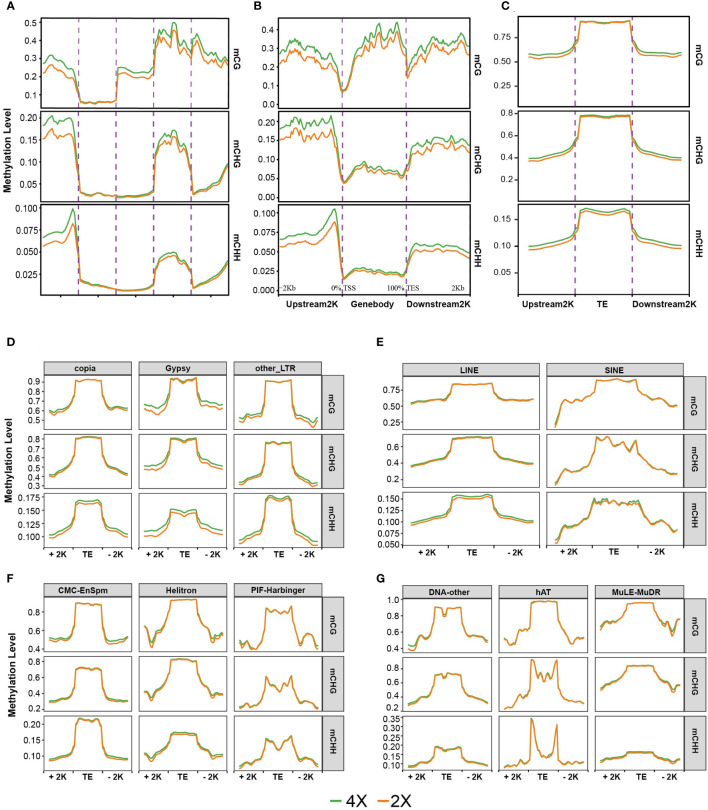
DNA methylation profiles. **(A)** Average methylation level in different regions of the genome. **(B)** Average methylation level distribution over gene body and flanking region. **(C)** Average methylation level distribution over TE and flanking region. **(D, E)** Average methylation level distribution over class I TEs. **(F, G)** Average methylation level distribution over class II TEs.

To characterize the DNA methylation patterns in the functional regions of different ploidies of water spinach, methylation profiles were constructed by counting the average methylation levels of C sites in each context in various genomic functional regions. The methylation levels had a similar trend in CHG and CHH contexts, with high methylation levels only in the promoter and intron regions. In contrast, exon regions had low methylation levels while intron regions had high methylation levels in CG context(**Figure 6A**). Further, the methylation levels in the upstream and downstream 2 kb flanking regions of the genes were analyzed. The results showed that there were a peak of CG methylation levels in the genebody regions, as well as two valleys near the transcription start and termination sites. The peak CG methylation in the Genebody was higher than that in the flanking region. In contrast, the methylation levels of CHH and CHG were significantly lower in the Genebody region than in the flanking regions (**Figure 6B**). The methylation levels were similar in all three contexts in 2X and 4X water spinach, but the methylation levels were higher in 4X water spinach than in 2X.

In our study, the average methylation level of TEs was much higher than that of genes, which was consistent with previous studies Notably, all three methylation contexts showed similar patterns, where TE bodies were highly methylated compared to upstream and downstream regions, and 4X water spinach had increased levels of CHG and CHH methylation in TE bodies relative to diploid (**Figure 6C**).

TEs were divided into two classes: Class I (retrotransposons) and Class II (DNA transposons). Next, methylation levels were analyzed for each methylation context in the 11 major orders. The results displayed that all types of TE have unique methylation profiles, the methylation levels of the body regions were higher than that of the flanking regions in all types of TEs. 4X water spinach exhibited hypermethylated levels in the CHG and CHH contexts in the body regions of Copia, Gypsy, and LINE, respectively. 4X water spinach showed hypermethylation levels in the flanking regions of LTR. Class II DNA transposons showed similar methylation levels in the two genotypes of water spinach. Among them, Helitron was highly methylated in the CHH context of 4X water spinach (**Figures 6D–G**).

### 3.5 Correlation of gene expression with DNA methylation and TE insertion

The comparison of the gene-expression level between different ploidies of water spinach from transcriptome data indicated that there were 971 differentially expressed genes, of which 475 were up-regulated and 496 were down-regulated compared to 2X water spinach. The results of GO enrichment analysis showed that the down-regulated genes were mainly enriched in the BP process, ‘cellular carbohydrate metabolic process’ and ‘disaccharide metabolic process’ were the most enriched in the BP categories, ‘cell wall’ and ‘external encapsulating structure’ were the most enriched in the CC categories. In the MF category, the subcategory with the highest enrichment degree was ‘amino acid binding’, followed by ‘carboxylic acid binding’ and ‘organic acid binding’. KEGG analysis of transcriptomics showed that DEGs had significantly enriched in ‘Fatty acid high-temperature’, ‘Steroid biosynthesis’, ‘Circadian rhythm – plant’, ‘Starch and sucrose metabolism’ pathways (**Figure S1**).

**Figure 7:**
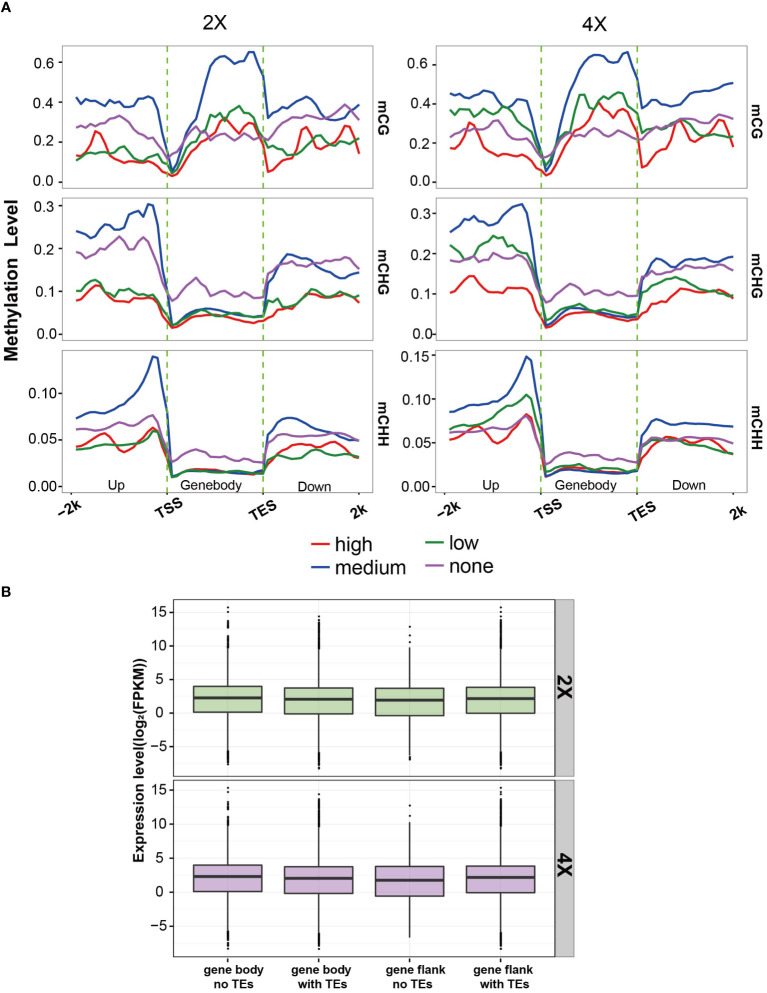
Correlation of gene expression with DNA methylation and TE insertion. **(A)** Association between DNA methylation level and gene expression in 2X and 4X water spinach. **(B)** The expression level of genes with or without TE insertion.

Based on the difference in methylation between 2X and 4X water spinach after genome doubling, the study tried to understand whether gene expression levels were affected by DNA methylation. Therefore, a total of 24,138 genes from RNA-seq data were classified into four quartiles of the none-expressed, low-expressed, medium-expressed, and high-expressed groups according to gene expression levels. In the genebody regions, the highest mCG methylation levels were not detected in the high-expressed genes, but were detected in those with medium-expressed, and none-expressed genes showed the lowest mCG methylation levels in 2X and 4X water spinach. There was a similar correlation between mCHH and mCHG methylation levels and gene expression, with high-expressed genes displaying the lowest methylation levels, and none-expressed genes displayed the highest methylation levels in the genebody regions. The results showed that mCG levels in the genebody region were positively correlated with gene expression levels, while CHG and CHH methylation levels were negatively correlated with gene expression in 2X and 4X water spinach (**Figure 7A**).

Then, the relationship between TE insertion and gene expression was analyzed. In autotetraploid and diploid water spinach, the expression level of genes inserted with TE was lower than those without TE insertion, but the expression level of genes inserted TE with flanking was higher than those without TE insertion. These results indicated that the gene expression level was affected by TE insertion (**Figure 7B**).

### 3.6 Differential methylation regions between 2X and 4X water spinach

**Figure 8:**
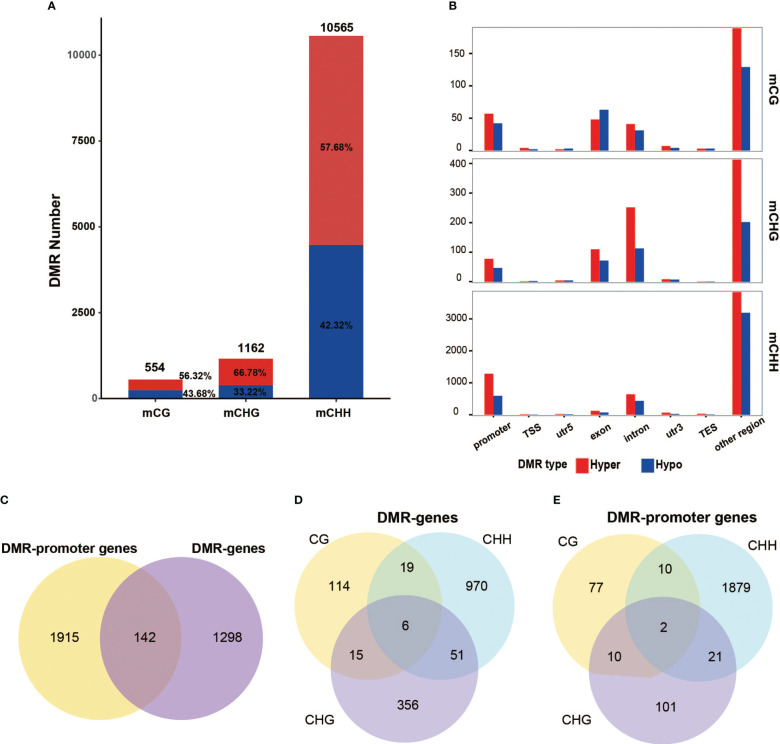
Differential methylome analysis for 4X water spinach compared with 2X. **(A)** Number of differentially hypermethylated and hypomethylated regions in all three contexts. **(B)** The distribution of DMRs in the genome. **(C)** Venn diagram of DMR-genes and DMR-promoter genes. **(D)** Venn diagrams of DMR-genes in all three contexts. **(E)** Venn diagrams of DMR-promoter genes in all three contexts.

To investigate DNA methylation variation in specific regions, the differential methylation regions (DMRs) were analyzed between 2X and 4X water spinach. In total, 554 CG, 1,162 CHG, and 10,565 CHH DMRs were identified with most of the DMRs from the CHH context genome-wide. Then, the DMRs were distinguished between hypermethylated DMR (hyper) and hypomethylated DMR (hypo), and the results showed that the number of hyper-DMRs was higher than that of hypo-DMRs for 4X compared with 2X (**Figure 8A**). The DMRs were inclined to localize the promoter regions rather than the gene body regions in CHH context, whereas, in the CG and CHG contexts, DMRs were distributed in the promoter, exon, and intron regions (**Figure 8B**). DMR identification was performed in three contexts separately, and the anchored genes (from TSS to TES), and anchored promoter region-related genes were plotted in the Venn diagram. The results showed that only a few DMR-genes and DMR-promoter genes exist in different contexts simultaneously, and most of them were discovered respectively (**Figures 8D, E**). In total, 3355 genes (DMR-related genes) were covered by 12281 DMRs, including 1440 DMR genes and 2057 DMR-promoter genes. Venn diagram showed that 142 genes had DMRs in both promoter and genebody regions. (**Figure 8C**).

**Figure 9:**
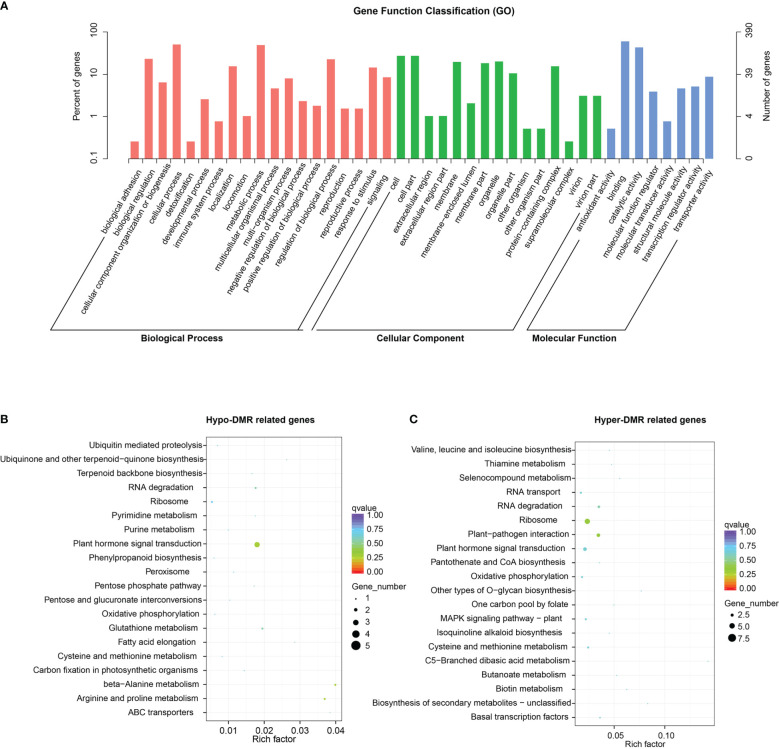
Enrichment analysis of DMR-related genes. **(A)** Gene ontology (GO) categories enriched in DMR-related genes. **(B, C)** KEGG enrichment of hypo-DMR-related genes and hyper- DMR-related genes.

To determine which biological processes and metabolic pathways were associated with DMRs, 3355 DMR-related genes were enriched and analyzed. Gene ontology (GO) analysis indicated that they were involved in the ‘cellular process’, ‘regulation of biological process’, ‘regulation of biological process’, ‘responses to stimuli’, ‘metabolic process’, and other processes (**Figure 9A**).

KEGG pathway analyses showed that hypo-DMR-related genes were enriched in ‘Arginine and proline metabolism’, ‘beta−Alanine metabolism’, ‘RNA degradation’, and ‘Plant hormone signal transduction’ pathways. Hyper-DMR-related genes showed enrichment in ‘Ribosome’, ‘Plant−pathogen interaction’, ‘Basal transcription factors’ and ‘Biotin metabolism’ pathways. Notably, both hypermethylated and hypomethylated DMR-related genes were enriched in ‘Plant−pathogen interaction’ and ‘Ribosome’ pathways (**Figures 9B, C**).

### 3.7 Combined analysis of DMR-related genes and DEGs

**Figure 10:**
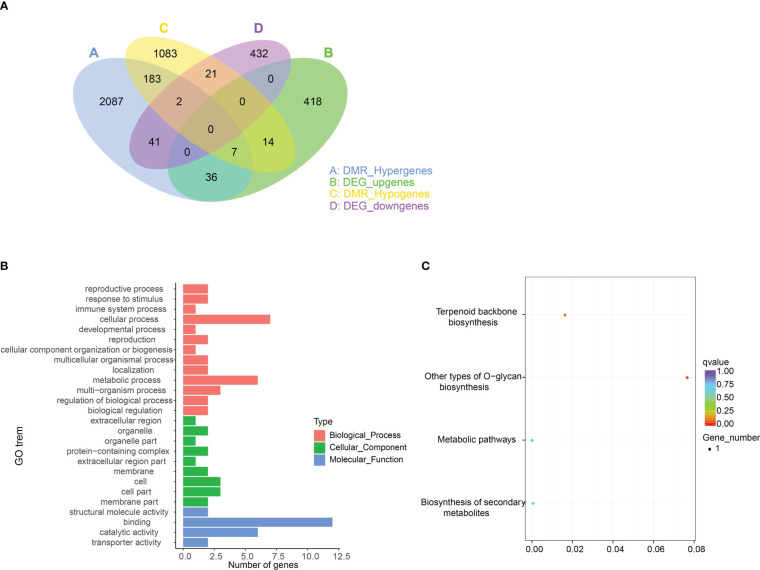
Combined analysis of DMR-related genes and DEGs. **(A)** Venn diagram of DMR-related genes and DEGs. **(B)** GO enrichment of 121 overlap genes. **(C)** KEGG enrichment of 121 overlapping genes.

To assess the relationship between methylation changes and transcription changes, the overlapping genes between DEGs and DMR-related genes were identified. A total of 121 genes overlapped between DEGs and DMR-related genes, among which 43 genes were hypermethylated with downregulated expression levels, and 21 genes were hypomethylated with upregulated expression levels in 4X vs 2X (**Table S5**). These genes may have negative regulatory mechanisms of methylation levels and gene expression. However, 43 upregulated and 23 downregulated genes were hypermethylated and hypomethylated, respectively (**Figure 10A**). These findings showed that in most cases, gene expression may not be associated with differences in methylation levels.

Furthermore, 121 overlapping genes were enriched, and GO enrichment showed they were mainly involved in ‘cellular process’, ‘metabolic process’, ‘binding’, and ‘catalytic activity’ terms. At the same time, these overlapping genes were related to the four pathways of ‘Other types of O-glycan biosynthesis’, ‘Terpenoid backbone biosynthesis’, ‘Biosynthesis of secondary metabolites’, and ‘Metabolic pathways’ (**Figures 10B, C**).

## 4 Discussion

Polyploidy was usually accompanied by morphological variation, polyploids showed more vigor and biomass in general compared to their diploid counterparts (Zhang et al., 2015; Hu et al., 2021). Polyploid plants usually increase their cell size, consequently developing large organs (Ding and Chen, 2018). The results of the present study showed autotetraploid water spinach plants had larger leaves, thicker stems, and larger stomata than the diploid parent, probably due to cell expansion caused by polyploid events as evident in previous studies (Kondorosi et al., 2000; Wang et al., 2019; Catalano et al., 2021; Wen et al., 2022). At the same time, the results of microscopic observation showed that the autotetraploid water spinach had larger spongy tissue, palisade tissue, and xylem cells. In addition, the previous study has shown that polyploidy causes morphological variation and changes flowering time (Yan et al., 2019), which needs further investigation in water spinach.

Genome doubling is usually accompanied by changes in DNA methylation. DNA methylation is an important mechanism in most biological processes, including plant growth and development, fruit ripening, and stress response (Huang et al., 2019; Lloyd and Lister, 2022). Previous studies revealed that genome size is positively correlated with the DNA methylation level in CG and CHG contexts but not in the CHH context (Niederhuth et al., 2016; Xu et al., 2018). This study revealed genome-wide changes in DNA methylation by WGBS sequencing technology and confirmed that genome doubling could induce genome-wide changes in DNA methylation, the methylation level of water spinach was moderate among species. In addition, the methylation level of 4X water spinach was higher than that of its diploid parent, which may be due to the expansion of the autotetraploid genome caused by genome duplication. In this study, more than half of the mCs came from CHH context, while more mCs appeared in CG context of grape and *Arabidopsis* (Zhang et al., 2015; Xiang et al., 2023). This indicates that there was a unique DNA methylation pattern in water spinach.

Previous studies have suggested that methylation of the gene body region is exclusive to angiosperms (Bewick and Schmitz, 2017). In this study, the genebody region showed hypermethylation only in CG context, while it showed hypomethylation in CHH and CHG contexts. DNA methylation levels in TE are significantly higher than in the genebody, which was consistent with studies of rice (Zhang et al., 2015), grape (Xiang et al., 2023) and switchgrass (Yan et al., 2019). These results indicated that DNA methylation was closely related to plant biological processes.

DNA methylation not only maintains genomic stability but also helps regulate gene expression. The relationship between transcription and DNA methylation is complex (Seymour and Becker, 2017). Genic methylation is strongly influenced by transcription: moderately transcribed genes are hypermethylated, whereas genes at either extreme are least likely to be methylated (Zilberman et al., 2007). Promoter methylation is usually associated with gene suppression or silencing (Li et al., 2012), but the opposite situation also exists (Ding et al., 2022). Here, in autotetraploid water spinach, the high-expressed genes had low methylation levels, the medium-expressed genes have the highest methylation level in promoter region, low-expressed genes had higher methylation levels than none-expressed genes. These results suggested that methylation levels in the promoter region regulate gene expression after genome doubling. However, DNA methylation studies in many species have shown that genebody methylation level seems to be positively correlated with gene expression in CG context, whereas methylation in non-CG contexts was negatively correlated with gene expression (Hu et al., 2014; Xu et al., 2018; Xiao et al., 2022). Transposable elements (TEs) are mobile genetic elements that are ubiquitous in plant genomes and silenced by epigenetic modifications. They are generally divided into retrotransposons (class I) and DNA transposons (class II) (Feschotte et al., 2002; Underwood et al., 2017). TEs are typically silenced by epigenetic mechanisms such as histone modifications and DNA methylation, with adverse effects on the expression of nearby genes (Bucher et al., 2012; Cui and Cao, 2014; Le et al., 2015). Previous studies have confirmed the complex correlation between TE and DNA methylation (Inagaki, 2022; Ramakrishnan et al., 2022). The insertion of TEs within or close proximity to genes can disrupt gene expression, producing negative phenotypic and fitness consequences(Bewick and Schmitz, 2017). In the present study, the number, distance, and methylation status of TE affected neighboring gene expression(Hollister and Gaut, 2009; Hollister et al., 2011). This study revealed that This study showed that the gene expression was influenced by TE insertion or not. TE insertion in the gene body inhibited gene expression, while TE insertion in the flanking region activated gene expression.

In addition, a total of 554 CG, 1162 CHG and 10565 CHH DMRs were identified after genome doubling, among which were more hyper-DMRs. In the present study, more hypo-DMRs were produced after whole-genome double(Yan et al., 2019; Xiang et al., 2023). Moreover, in our study, the DMRs mainly come from CHH context, which is inconsistent with grape, switchgrass, and cassava(Xiao et al., 2022), and consistent with rice(Zhang et al., 2015). These results indicated that different methylation patterns exist in different species after genome-wide doubling. DNA hypermethylation may be one of the important processes regulating phenotypic changes after genome doubling. 12,281 DMRs covered 3,355 genes (DMR-related genes), and the enrichment analysis showed that they were involved in “cellular process”, “biological process regulation”, “biological process regulation”, “stimulus response”, “metabolic process” and other processes. In this study, 121 genes overlapped between DEG and DMR-related genes, which are related to signal transduction and growth and development. This suggests that the majority of DMRs were connected with phenotypic variations rather than having a direct impact on gene expression. The present study confirms the existence of indicative differences between autotetraploid and diploid water spinach, and this variation was closely related to DNA methylation.

## 5 Conclusions

In this study, autotetraploid water spinach was successfully created by colchicine treatment, and genome doubling caused obvious morphological variation of autotetraploid water spinach. Genome doubling was accompanied by an increase in DNA methylation level. Autopolyploidization affected the expression levels of nearby genes by inducing genome-wide variation in DNA methylation. A total of 12281 DMRs were identified, and 3355 DMR-related genes were covered. These genes are mainly involved in the metabolism and regulation of biological processes in plants. A total of 121 genes overlapped between DEGs and DMR-related genes, they were believed to be critical genes involved in regulating gene expression through DNA methylation. The present results preliminarily reveal the apparent mechanism of phenotypic variation in autotetraploid water spinach. In the following work, we can further investigate the mechanism of DNA methylation affecting gene expression.

## Data availability statement

The datasets presented in this study can be found in online repositories. The names of the repository/repositories and accession number(s) can be found in the article/**Supplementary Material**.

## Author contributions

Conceptualization, ZW and YH. Methodology, ZW, YH, XS, WL, MM. Investigation, YH, HS, LL, YZ, SC, and GZ. Writing—original draft preparation, YH. Supervision, ZW. All authors contributed to the article and approved the submitted version.

## References

[B1] Abdolinejad R. Shekafandeh A. (2022). Tetraploidy confers superior *in vitro* water-stress tolerance to the fig tree (*Ficus carica*) by reinforcing hormonal, physiological, and biochemical defensive systems. Front. Plant Sci. 12. doi: 10.3389/fpls.2021.796215 PMC8834540 35154187

[B2] Ahmad R. Anjum M. A. (2018). Applications of molecular markers to assess genetic diversity in vegetable and ornamental crops – a review. Hortic. Sci. Technol. 1, 1–7. doi: 10.46653/jhst180101001

[B3] Allario T. Brumos J. Colmenero-Flores J. M. Tadeo F. Froelicher Y. Talon M.. (2011). Large Changes in anatomy and physiology between diploid rangpur lime (*Citrus limonia*) and its autotetraploid are not associated with large changes in leaf gene expression. J. Exp. Bot. 62 (8), 2507–2519. doi: 10.1093/jxb/erq467 21273338

[B4] Altaf M. A. Shahid R. Ren M. X. Naz S. Hayat F. (2020). Exogenous melatonin enhances salt stress tolerance in tomato seedlings. Biol. Plantar 64, 604–615. doi: 10.32615/bp.2020.090

[B5] Bewick A. J. Schmitz R. J. (2017). Gene body DNA methylation in plants. Curr. Opin. Plant Biol. 36, 103–110. doi: 10.1016/j.pbi.2016.12.007 28258985 PMC5413422

[B6] Bucher E. Reinders J. Mirouze M. (2012). Epigenetic control of transposon transcription and mobility in *Arabidopsis* . Curr. Opin. Plant Biol. 15 (5), 503–510. doi: 10.1016/j.pbi.2012.08.006 22940592

[B7] Catalano C. Abbate L. Motisi A. Crucitti D. Cangelosi V. Pisciotta A.. (2021). Autotetraploid emergence *via* somatic embryogenesis in vitis vinifera induces marked morphological changes in shoots, mature leaves, and stomata. Cells 10 (6), 1336. doi: 10.3390/cells10061336 34071294 PMC8228502

[B8] Chen Z. J. (2007). Genetic and epigenetic mechanisms for gene expression and phenotypic variation in plant polyploids. Annu. Rev. Plant Biol. 58, 377–406. doi: 10.1146/annurev.arplant.58.032806.103835 17280525 PMC1949485

[B9] Comai L. (2005). The advantages and disadvantages of being polyploid. Nat. Rev. Genet. 6 (11), 836–846. doi: 10.1038/nrg1711 16304599

[B10] Cui X. Cao X. (2014). Epigenetic regulation and functional exaptation of transposable elements in higher plants. Curr. Opin. Plant Biol. 21, 83–88. doi: 10.1016/j.pbi.2014.07.001 25061895

[B11] Dai F. Wang Z. Luo G. Tang C. (2015). Phenotypic and transcriptomic analyses of autotetraploid and diploid mulberry (*Morus alba* l.). Int. J. Mol. Sci. 16 (9), 22938–22956. doi: 10.3390/ijms160922938 26402678 PMC4613344

[B12] del Pozo J. C. Ramirez-Parra E. (2014). Deciphering the molecular bases for drought tolerance in *Arabidopsis* autotetraploids. Plant Cell Environ. 37 (12), 2722–2737. doi: 10.1111/pce.12344 24716850

[B13] Ding M. Chen Z. J. (2018). Epigenetic perspectives on the evolution and domestication of polyploid plant and crops. Curr. Opin. Plant Biol. 42, 37–48. doi: 10.1016/j.pbi.2018.02.003 29502038 PMC6058195

[B14] Ding Y. Q. Zou L. H. Wu J. J. Ramakrishnan M. Gao Y. B. Zhao L. Z.. (2022). The pattern of DNA methylation alteration, and its association with the expression changes of non-coding RNAs and mRNAs in *Moso bamboo* under abiotic stress. Plant Sci. 325, 111451. doi: 10.1016/j.plantsci.2022.111451 36075278

[B15] Dpooležel J. Binarová P. Lcretti S. (1989). Analysis of nuclear DNA content in plant cells by flow cytometry. Biol. Plantar 31 (2), 113–120. doi: 10.1007/BF02907241

[B16] Feschotte C. Jiang N. Wessler S. R. (2002). Plant transposable elements: where genetics meets genomics. Nat. Rev. Genet. 3 (5), 329–341. doi: 10.1038/nrg793 11988759

[B17] Gupta C. Salgotra R. K. (2022). Epigenetics and its role in effecting agronomical traits. Front. Plant Sci. 13. doi: 10.3389/fpls.2022.925688 PMC9421166 36046583

[B18] Hegarty M. Coate J. Sherman-Broyles S. Abbott R. Hiscock S. Doyle J. (2013). Lessons from natural and artificial polyploids in higher plants. Cytogenet. Genome Res. 140 (2-4), 204–225. doi: 10.1159/000353361 23816545

[B19] Hewitson T. D. Wigg B. Becker G. J. (2010). Tissue preparation for histochemistry: fixation, embedding, and antigen retrieval for light microscopy. Methods Mol. Biol. 611, 3–18. doi: 10.1007/978-1-60327-345-9_1 19960318

[B20] Hollister J. D. Gaut B. S. (2009). Epigenetic silencing of transposable elements: a trade-off between reduced transposition and deleterious effects on neighboring gene expression. Genome Res. 19 (8), 1419–1428. doi: 10.1101/gr.091678.109 19478138 PMC2720190

[B21] Hollister J. D. Smith L. M. Guo Y. L. Ott F. Weigel D. Gaut B. S. (2011). Transposable elements and small RNAs contribute to gene expression divergence between *Arabidopsis thaliana* and *Arabidopsis lyrata* . Proc. Natl. Acad. Sci. U.S.A. 108 (6), 2322–2327. doi: 10.1073/pnas.1018222108 21252301 PMC3038775

[B22] Hu L. Li N. Xu C. Zhong S. Lin X. Yang J.. (2014). Mutation of a major CG methylase in rice causes genome-wide hypomethylation, dysregulated genome expression, and seedling lethality. Proc. Natl. Acad. Sci. U. S. A. 111 (29), 10642–10647. doi: 10.1073/pnas.1410761111 25002488 PMC4115543

[B23] Hu Y. Sun D. Hu H. Zuo X. Xia T. Xie J. (2021). A comparative study on morphological and fruit quality traits of diploid and polyploid carambola (*Averrhoa carambola* l.) genotypes. Sci. Hortic-Amsterdam 277, 109843. doi: 10.1016/j.scienta.2020.109843

[B24] Huang H. Liu R. Niu Q. Tang K. Zhang B. Zhang H.. (2019). Global increase in DNA methylation during orange fruit development and ripening. Proc. Natl. Acad. Sci. U. S. A. 116 (4), 1430–1436. doi: 10.1073/pnas.1815441116 30635417 PMC6347674

[B25] Inagaki S. (2022). Silencing and anti-silencing mechanisms that shape the epigenome in plants. Genes Genet. Syst. 96 (5), 217–228. doi: 10.1266/ggs.21-00041 34719532

[B26] Jiang X. Song Q. Ye W. Chen Z. J. (2021). Concerted genomic and epigenomic changes accompany stabilization of *Arabidopsis* allopolyploids. Nat. Ecol. Evol. 5 (10), 1382–1393. doi: 10.1038/s41559-021-01523-y 34413505 PMC8484014

[B27] Kondorosi E. Roudier F. Gendreau E. (2000). Plant cell-size control: growing by ploidy? Curr. Opin. Plant Biol. 3 (6), 488–492. doi: 10.1016/s1369-5266(00)00118-7 11074380

[B28] Kuo J. (2014). Processing plant tissues for ultrastructural study. Methods Mol. Biol. 1117, 39–55. doi: 10.1007/978-1-62703-776-1_3 24357358

[B29] Le T. N. Miyazaki Y. Takuno S. Saze H. (2015). Epigenetic regulation of intragenic transposable elements impacts gene transcription in *Arabidopsis thaliana* . Nucleic Acids Res. 43 (8), 3911–3921. doi: 10.1093/nar/gkv258 25813042 PMC4417168

[B30] Li X. Zhu J. D. Hu F. Y. Ge S. Ye M. Z. Xiang H.. (2012). Single-base resolution maps of cultivated and wild rice methylomes and regulatory roles of DNA methylation in plant gene expression. BMC Genomics 13, 300. doi: 10.1186/1471-2164-13-300 22747568 PMC3447678

[B31] Lloyd J. P. B. Lister R. (2022). Epigenome plasticity in plants. Nat. Rev. Genet. 23 (1), 55–68. doi: 10.1038/s41576-021-00407-y 34526697

[B32] Ma Y. Xue H. Zhang L. Zhang F. Ou C. Wang F.. (2016). Involvement of auxin and brassinosteroid in dwarfism of autotetraploid apple (*Malus* × *domestica*). Sci. Rep. 6, 26719. doi: 10.1038/srep26719 27216878 PMC4877651

[B33] Madlung A. Wendel J. F. (2013). Genetic and epigenetic aspects of polyploid evolution in plants. Cytogenet. Genome Res. 140 (2-4), 270–285. doi: 10.1159/000351430 23751292

[B34] Miura F. Enomoto Y. Dairiki R. Ito T. (2012). Amplification-free whole-genome bisulfite sequencing by post-bisulfite adaptor tagging. Nucleic Acids Res. 40 (17), e136. doi: 10.1093/nar/gks454 22649061 PMC3458524

[B35] Nassar N. M. Graciano-Ribeiro D. Fernandes S. D. Araujo P. C. (2008). Anatomical alterations due to polyploidy in cassava, *Manihot esculenta* crantz. Genet. Mol. Res. 7 (2), 276–283. doi: 10.4238/vol7-2gmr399 18551393

[B36] Niederhuth C. E. Bewick A. J. Ji L. Alabady M. S. Kim K. D. Li Q.. (2016). Widespread natural variation of DNA methylation within angiosperms. Genome Biol. 17 (1), 194. doi: 10.1186/s13059-016-1059-0 27671052 PMC5037628

[B37] Pathan A. K. Bond J. Gaskin R. E. (2008). Sample preparation for scanning electron microscopy of plant surfaces–horses for courses. Micron 39 (8), 1049–1061. doi: 10.1016/j.micron.2008.05.006 18586502

[B38] Qin J. Mo R. Li H. Ni Z. Sun Q. Liu Z. (2021). The transcriptional and splicing changes caused by hybridization can be globally recovered by genome doubling during allopolyploidization. Mol. Biol. Evol. 38 (6), 2513–2519. doi: 10.1093/molbev/msab045 33585937 PMC8136492

[B39] Qiu T. Liu Z. Liu B. (2020). The effects of hybridization and genome doubling in plant evolution *via* allopolyploidy. Mol. Biol. Rep. 47 (7), 5549–5558. doi: 10.1007/s11033-020-05597-y 32572735

[B40] Ramakrishnan M. Satish L. Sharma A. Kurungara Vinod K. Emamverdian A. Zhou M.. (2022). Transposable elements in plants: Recent advancements, tools and prospects. Plant Mol. Biol. Rep. 40, 628–645. doi: 10.1007/s11105-022-01342-w

[B41] Ren R. Wang H. Guo C. Zhang N. Zeng L. Chen Y.. (2018). Widespread whole genome duplications contribute to genome complexity and species diversity in angiosperms. Mol. Plant 11 (3), 414–428. doi: 10.1016/j.molp.2018.01.002 29317285

[B42] Salmon A. Ainouche M. L. (2010). Polyploidy and DNA methylation: new tools available. Mol. Ecol. 19 (2), 213–225. doi: 10.1111/j.1365-294X.2009.04461.x 20078770

[B43] Seymour D. K. Becker C. (2017). The causes and consequences of DNA methylome variation in plants. Curr. Opin. Plant Biol. 36, 56–63. doi: 10.1016/j.pbi.2017.01.005 28226269

[B44] Syngelaki E. Paetzold C. Hörandl E. (2021). Gene expression profiles suggest a better cold acclimation of polyploids in the alpine species *Ranunculus kuepferi* (Ranunculaceae). Genes (Basel) 12 (11), 1818. doi: 10.3390/genes12111818 34828424 PMC8625111

[B45] Tayalé A. Parisod C. (2013). Natural pathways to polyploidy in plants and consequences for genome reorganization. Cytogenet. Genome Res. 140 (2-4), 79–96. doi: 10.1159/000351318 23751271

[B46] Tossi V. E. Martínez Tosar L. J. Laino L. E. Iannicelli J. Regalado J. J. Escandón A. S.. (2022). Impact of polyploidy on plant tolerance to abiotic and biotic stresses. Front. Plant Sci. 13. doi: 10.3389/fpls.2022.869423 PMC9441891 36072313

[B47] Touchell D. H. Palmer I. E. Ranney T. G. (2020). *In vitro* ploidy manipulation for crop improvement. Front. Plant Sci. 11. doi: 10.3389/fpls.2020.00722 PMC7284393 32582252

[B48] Underwood C. J. Henderson I. R. Martienssen R. A. (2017). Genetic and epigenetic variation of transposable elements in *Arabidopsis* . Curr. Opin. Plant Biol. 36, 135–141. doi: 10.1016/j.pbi.2017.03.002 28343122 PMC5746046

[B49] Vanyushin B. F. (2006). DNA Methylation in plants. Curr. Top. Microbiol. Immunol. 301, 67–122. doi: 10.1007/3-540-31390-7_4 16570846

[B50] Vanyushin B. F. Ashapkin V. V. (2011). DNA Methylation in higher plants: past, present and future. Biochim. Biophys. Acta 1809 (8), 360–368. doi: 10.1016/j.bbagrm.2011.04.006 21549230

[B51] Wang Y. Huang S. Liu Z. Tang X. Feng H. (2018). Changes in endogenous phytohormones regulated by microRNA-target mRNAs contribute to the development of dwarf autotetraploid Chinese cabbage (*Brassica rapa* l. ssp. pekinensis). Mol. Genet. Genomics 293 (6), 1535–1546. doi: 10.1007/s00438-018-1480-z 30116946

[B52] Wang L. Luo Z. Wang L. Deng W. Wei H. Liu P.. (2019). Morphological, cytological and nutritional changes of autotetraploid compared to its diploid counterpart in Chinese jujube (*Ziziphus jujuba* mill.). Sci. Hortic-Amsterdam 249, 263–270. doi: 10.1016/j.scienta.2019.01.063

[B53] Wen Y. Liu H. Meng H. Qiao L. Zhang G. Cheng Z. (2022). *In vitro* induction and phenotypic variations of autotetraploid garlic (*Allium sativum* l.) with dwarfism. Front. Plant Sci. 13. doi: 10.3389/fpls.2022.917910 PMC9258943 35812906

[B54] Xiang X. Gao Y. Cui J. Ren G. Yin C. Chang J. (2023). Methylome and transcriptome analysis of alters leaf phenotype with autotetraploid in grape. Sci. Hortic-Amsterdam 307, 111534. doi: 10.1016/j.scienta.2022.111534

[B55] Xiao L. Lu L. Zeng W. Shang X. Cao S. Yan H. (2022). DNA Methylome and LncRNAome analysis provide insights into mechanisms of genome-dosage effects in autotetraploid cassava. Front. Plant Sci. 13. doi: 10.3389/fpls.2022.915056 PMC9289687 35860527

[B56] Xu Y. Xu H. Wu X. Fang X. Wang J. (2012). Genetic changes following hybridization and genome doubling in synthetic *Brassica napus* . Biochem. Genet. 50 (7-8), 616–624. doi: 10.1007/s10528-012-9505-5 22538518

[B57] Xu Y. Zhong L. Wu X. Fang X. Wang J. (2009). Rapid alterations of gene expression and cytosine methylation in newly synthesized *Brassica napus* allopolyploids. Planta 229 (3), 471–483. doi: 10.1007/s00425-008-0844-8 18998158

[B58] Xu J. Zhou S. Gong X. Song Y. van Nocker S. Ma F.. (2018). Single-base methylome analysis reveals dynamic epigenomic differences associated with water deficit in apple. Plant Biotechnol. J. 16 (2), 672–687. doi: 10.1111/pbi.12820 28796917 PMC5787839

[B59] Yan H. Bombarely A. Xu B. Wu B. Frazier T. P. Zhang X.. (2019). Autopolyploidization in switchgrass alters phenotype and flowering time *via* epigenetic and transcription regulation. J. Exp. Bot. 70 (20), 5673–5686. doi: 10.1093/jxb/erz325 31419288

[B60] Zhang J. Liu Y. Xia E. H. Yao Q. Y. Liu X. D. Gao L. Z. (2015). Autotetraploid rice methylome analysis reveals methylation variation of transposable elements and their effects on gene expression. Proc. Natl. Acad. Sci. U. S. A. 112 (50), E7022–E7029. doi: 10.1073/pnas.1515170112 26621743 PMC4687577

[B61] Zhang K. Wang X. Cheng F. (2019). Plant polyploidy: origin, evolution, and its influence on crop domestication. Hortic. Plant J. 5 (6), 231–239. doi: 10.1016/j.hpj.2019.11.003

[B62] Zilberman D. Gehring M. Tran R. K. Ballinger T. Henikoff S. (2007). Genome-wide analysis of *Arabidopsis thaliana* DNA methylation uncovers an interdependence between methylation and transcription. Nat. Genet. 39 (1), 61–69. doi: 10.1038/ng1929 17128275

