# Supplementary material for: The creation of autotetraploid provides insights into critical features of DNA methylome changes after genome doubling in water spinach (Ipomoea aquatica Forsk)
Source: Front Plant Sci. 2023 Apr 14;14:1155531. doi: 10.3389/fpls.2023.1155531 (PMC10140364; doi:10.3389/fpls.2023.1155531)
Supplement: Supplementary file 1 [file DataSheet_1.docx]

Supplementary Material

Creation and DNA methylation analysis of autotetraploid of water spinach (*Ipomoea aquatica*)

Yuanyuan Hao*, Xiao Su, Wen Li, Lin Li, Yu Zhang, Muhammad Ali Mumtaz1, Huangying Shu, Shanhan Cheng, Guopeng Zhu,

*** Correspondence:** Zhiwei Wang: wangzhiwei@hainanu.edu.cn


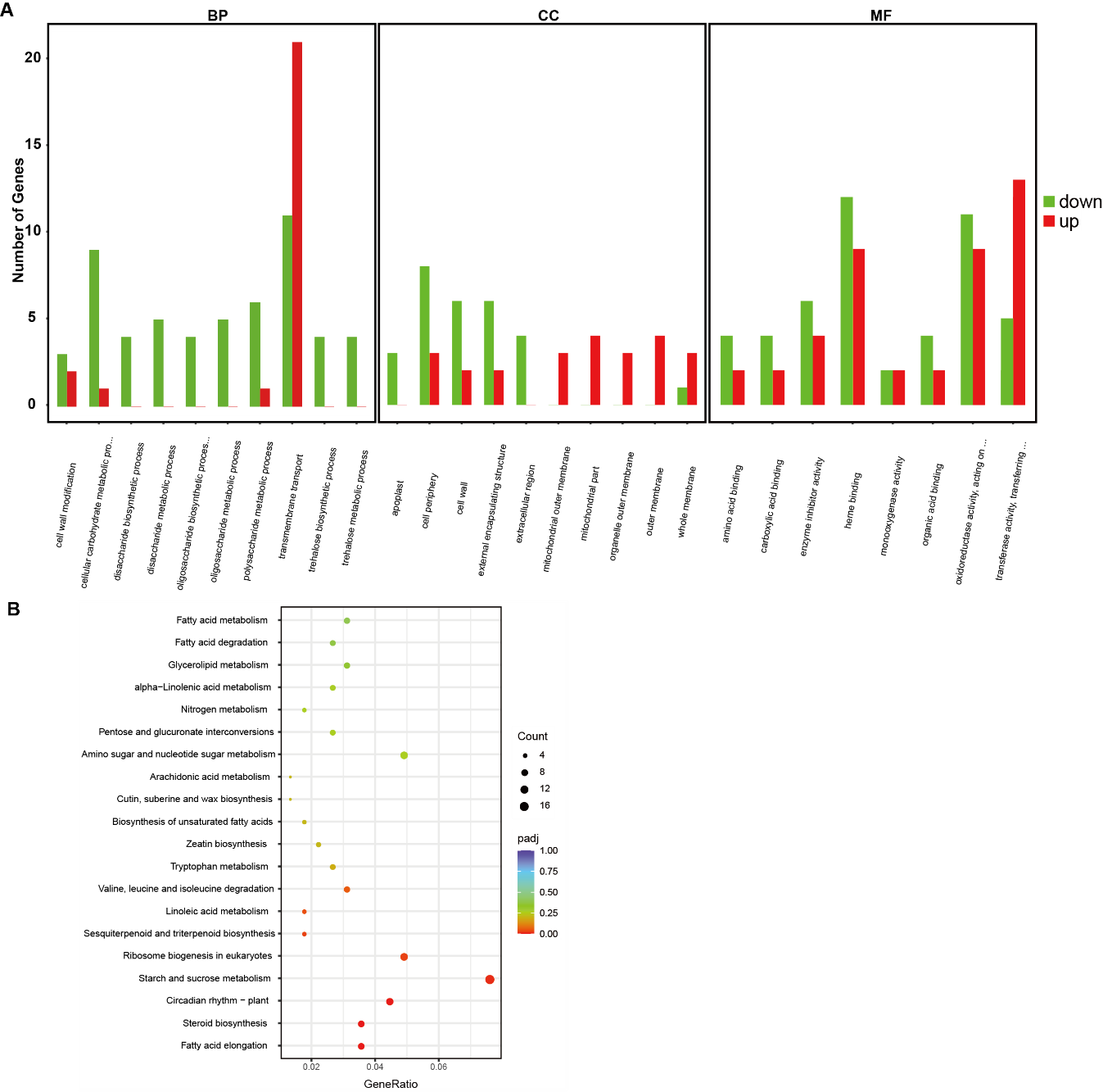


**Supplementary Figure 1.** DEGs enrichment analysis. (A): GO enrichment. (B) KEGG pathway enrichment.
